# Rhizophoraceae Mangrove Saplings Use Hypocotyl and Leaf Water Storage Capacity to Cope with Soil Water Salinity Changes

**DOI:** 10.3389/fpls.2016.00895

**Published:** 2016-06-27

**Authors:** Silvia Lechthaler, Elisabeth M. R. Robert, Nathalie Tonné, Alena Prusova, Edo Gerkema, Henk Van As, Nico Koedam, Carel W. Windt

**Affiliations:** ^1^Department of Territorio e Sistemi Agro-Forestali, University of Padova, PadovaItaly; ^2^Laboratory of Plant Biology and Nature Management, Department of Biology, Vrije Universiteit Brussel, BrusselsBelgium; ^3^Laboratory of Wood Biology and Xylarium, Department of Wood Biology, Royal Museum for Central AfricaTervuren, Belgium; ^4^Laboratory of Biophysics and Wageningen NMR Centre, Department of Agrotechnology & Food Sciences, Wageningen University, WageningenNetherlands; ^5^IBG-2: Plant Sciences, Institute for Bio- and Geosciences, Forschungszentrum Jülich, JülichGermany

**Keywords:** dendrometers, leaf thickness variations, magnetic resonance imaging (MRI), mangrove environment, mobile nuclear magnetic resonance (NMR)

## Abstract

Some of the most striking features of Rhizophoraceae mangrove saplings are their voluminous cylinder-shaped hypocotyls and thickened leaves. The hypocotyls are known to serve as floats during seed dispersal (hydrochory) and store nutrients that allow the seedling to root and settle. In this study we investigate to what degree the hypocotyls and leaves can serve as water reservoirs once seedlings have settled, helping the plant to buffer the rapid water potential changes that are typical for the mangrove environment. We exposed saplings of two Rhizophoraceae species to three levels of salinity (15, 30, and 0–5‰, in that sequence) while non-invasively monitoring changes in hypocotyl and leaf water content by means of mobile NMR sensors. As a proxy for water content, changes in hypocotyl diameter and leaf thickness were monitored by means of dendrometers. Hypocotyl diameter variations were also monitored in the field on a *Rhizophora* species. The saplings were able to buffer rapid rhizosphere salinity changes using water stored in hypocotyls and leaves, but the largest water storage capacity was found in the leaves. We conclude that in *Rhizophora* and *Bruguiera* the hypocotyl offers the bulk of water buffering capacity during the dispersal phase and directly after settlement when only few leaves are present. As saplings develop more leaves, the significance of the leaves as a water storage organ becomes larger than that of the hypocotyl.

## Introduction

Mangrove trees grow in one of the most challenging environments that vascular plants have been able to colonize. Due to intermittent tidal and fluvial inundation, irregular freshwater input, and salt accumulation by evaporation, mangrove trees do not only have to deal with extreme, but also with highly variable levels of soil water salinity (Krauss and Ball, 2012; Bompy et al., 2014b). In addition, they experience hypoxic soils, periodic partial or entire inundation, large changes in VPD, and high levels of solar radiation (Ball, 1988a; Tomlinson, 1994; Robert et al., 2009). Mangroves are able to exclude salt when taking up water from their substrate (Scholander, 1968; Khan and Aziz, 2001; Parida and Jha, 2010) and/or excrete it from their leaves (Wang et al., 2011). The exclusion of salt, however, is only partial and elevated salt concentrations are known to be tolerated in the cell apoplast (from 1 to 10% seawater salinity). Mangroves have been suggested to take advantage of elevated salt concentrations in the apoplast and symplast to decrease water potential, thus reducing the challenge to primary walls in vessels and to cell membranes (Scholander et al., 1966; Parida and Jha, 2010; Reef and Lovelock, 2015).

To be able to deal with these rapidly changing abiotic conditions, mangrove trees developed various means to store and buffer water. The ability to store water is not unique for salt tolerant plants; in many plants and trees it is associated with resistance to drought. The role of the stem as a water storage organ in trees has received considerable attention (Zweifel et al., 2000; Scholz et al., 2008; Carrasco et al., 2015; Morris et al., 2015; Pfautsch et al., 2015b). Various studies demonstrated that living tissues associated with the xylem are able to store water (paratracheal and apotracheal parenchyma, phloem, cambium, bark) and transport it axially (parenchyma rays), thus allowing the tree to buffer water potential changes in its abiotic environment (Spicer, 2014; Pfautsch et al., 2015a and references therein).

For mangrove trees, which usually have access to water but periodically may have difficulty to take it up because of its salinity, the most important benefit of water storage appears to be that it minimizes the carbon costs that are associated with water uptake, osmoregulation, and the upkeep of tissue salt tolerance. For example, the size of the mangrove root system has been shown to correlate with salt resistance: the higher the salt resistance, the larger the root system (Ball, 1988b; Sherman et al., 2003; Adame et al., 2014). The size of the root system thus is suggested to be the limiting factor for water uptake under elevated salinity (Ball, 1988a). Increasing root size, however, brings with it considerable carbon costs for the plant. First in growing the larger root system (Ball, 2002), but subsequently also in operating that root system. Salt exclusion during uptake requires significant amounts of energy (Ball, 1988a,b), and so does osmotic regulation (Munns and Gilliham, 2015). It has been argued that an increased salt tolerance thus comes at the expense of growth and competitive ability at low salinities (Ball, 2002).

Water storage capacity may help to minimize the direct and indirect costs of water uptake in two ways. Firstly, by spreading water uptake over the day and buffering peak demand. In this way the maximum water uptake per unit time is reduced, and so is the size of the root system that would be required to supply it. Secondly, by enabling the tree to take up water when it is less costly to do so: when salinities are low, at cooler temperatures with higher relative humidity (RH), during rain events, or at night when stomata are closed. Mangrove trees with an enhanced water storage capacity thus will be able to take up water more efficiently than those without, giving them a competitive advantage.

Larger trees have a larger relative water storage capacity and cope better with changes in water potential (Scholz et al., 2011 and references therein). It would then follow that small saplings have an especially difficult time to cope with such changes and could benefit greatly from specialized organs to provide additional water storage capacity. Mangrove saplings are especially likely to be confronted with strong salinity fluctuations, as without deep roots they will have to acquire water in the topsoil region where salinity will be the most variable (Bompy et al., 2014b). In such an environment, mangrove seedlings with an enhanced water storage capacity could have a competitive edge, which in turn could be an important determinant of forest structure and species composition (Smith and Snedaker, 1995; Ball, 2002; Bompy et al., 2014a).

**FIGURE 1:**
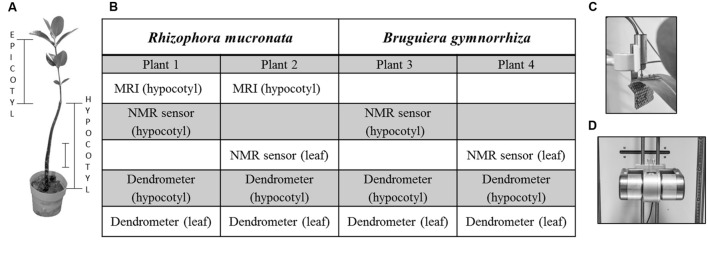
**(A)** Epicotyl and hypocotyl of a *Rhizophora mucronata* sapling. The scale bar represents 10 cm. **(B)** Table outlining which measurements were conducted per plant. **(C,D)** Dendrometers employed to measure variation in hypocotyl diameter and **(C)** in leaf thickness. **(D)** Continuous water content (WC_coil_) measurements were done by means of mobile small scale NMR sensors.

The hypocotyl is the basal enlarged part of the propagule that after settlement is found between the root and the cotyledons, particularly in Rhizophoraceae. Many mangroves are viviparous: the embryo develops without immediate dormancy and the developing propagule detaches from the ovary and disperses by floating (Tomlinson, 1994). Rhizophoraceae produce especially long and large propagules, which after settlement become voluminous cylindrical hypocotyls topped by a smaller epicotyl (**Figure 1A**). After serving its role as a float during hydrochorous dispersal, the hypocotyl supplies nutrients and carbohydrates to facilitate rooting and settlement (Bobda et al., 2014; Robert et al., 2015; Tonné et al., 2016). It typically has a length of 15–70 cm and a diameter of 0.5–3 cm (Tomlinson, 1994). Because of its first function as a buoy the hypocotyl is not expected to have a very high tissue density. However, its large volume, mainly occupied by parenchyma and aerenchyma tissues, does invite the question whether after settlement and expenditure of its stored carbohydrates (Ball, 2002), it could change function and also serve as a water storage organ.

An organ that is also known to be suitable for storing water is the leaf (which do not develop until after settlement). Leaves provide significant water storage capacity (Schulze et al., 1985; Sack and Tyree, 2005; Blackman and Brodribb, 2011), especially when they are succulent (Scholz et al., 2011; Ishii et al., 2014). In mangroves an increased tendency toward succulence and high leaf water content is observed at high salinities, as seen in *Avicennia germinans* (Suárez and Sobrado, 2000), *Laguncularia racemosa* (Sobrado, 2005), and *Bruguiera parviflora* (Parida et al., 2004). A larger leaf mass increases heat capacity, thus reducing the need for evaporative cooling (for example when leaves are transiently exposed to full sunlight), while at the same time increasing water storage capacity (Ball, 1988b; Suárez and Sobrado, 2000; Wang et al., 2011). This may allow leaves to keep their stomata open, even when water influx into the leaf cannot immediately keep up with transpiratory loss. Succulent leaves are suggested to enable mangrove plants to sequester large amounts of solutes without an adverse increase in cell osmotic pressure and to maintain turgor at low water potential (Suárez and Sobrado, 2000).

In this study we test whether the voluminous hypocotyl and the succulent leaves of Rhizophoraceae mangrove saplings can serve as water reservoirs to buffer sudden changes in soil water salinity. We hypothesize that for water storage tissues to have adaptive value, they should be able to contain and release a volume of water that is large enough to buffer such sudden changes. We distinguish short term water content fluctuations, understood as the volume of water that can be withdrawn from an organ during the day and replenished during the night (Goldstein et al., 1998), and long term water content fluctuations, the amount of water lost or gained due to prolonged exposure to changed abiotic conditions. To estimate the amount of water that the hypocotyls and leaves of *Rhizophora mucronata* and *Bruguiera gymnorrhiza* saplings have available to buffer changes in their abiotic environment, we challenged the plants with three step-wise soil salinity treatments lasting 5 days each: 15, 30, and 0–5‰ NaCl. During this period we monitored the water content of the hypocotyls and leaves by means of non-invasive mobile NMR sensors; changes in hypocotyl diameter and leaf thickness were measured with classical dendrometers. In addition, a field study on *Rhizophora mangle* saplings was done to test whether the use of hypocotyl water storage capacity could be detected under field conditions.

## Materials and Methods

### Plant Material

Four saplings of two mangrove species, *R. mucronata* (L.) LAMK (Rm – plants 1 and 2) and *B. gymnorrhiza* (L.) LAMK. (Bg – plants 3 and 4) were grown from propagules, collected in January 2013 in Mida Creek (Watamu Marine National Reserve, Kenya). After planting the propagules in regular potting soil and sand (1:1 ratio), they were grown at 15‰ NaCl in a greenhouse in Brussels, Belgium. During autumn and winter 100 μmol m^-2^ s^-1^ PAR of additional lighting was provided. Leaves were sprayed daily with freshwater to maintain the RH around 70%.

To elicit a release or uptake of water, *R. mucronata* and *B. gymnorrhiza* saplings were challenged with a three stage salinity treatment, 15 (native), 30, and 0–5‰, while continuously monitoring changes in the VWC of leaf and hypocotyl (by means of NMR), as well as changes in hypocotyl diameter and leaf thickness (by means of dendrometers).

### Magnetic Resonance Imaging Study

Hypocotyl water content was measured in *R. mucronata* plants 1 and 2 (**Figure 1B**) by means of nuclear MRI at the Wageningen NMR Centre (Wageningen University, The Netherlands). A 3T imager was employed, consisting of a 50 cm vertical bore magnet (Magnex, Oxford, UK) and a 1 T m^-1^ openable gradient coil (Bruker, Karlsruhe, Germany). A RF coil with an inner diameter of 4 cm was placed around the plant’s hypocotyl at a height of 28 cm. Inside the scanner the plants were exposed to an air temperature of between 22 and 24°C, a RH of 40% and a day time light intensity of 120 μmol m^-2^ s^-1^ PAR at leaf level. Between measurements the plants were grown in a climate chamber (20°C night and 26°C day; RH: 70%; 100 μmol m^-2^ s^-1^ PAR; 12 h photoperiod). The plants were sprayed with freshwater.

The CPMG measurements were done using the following settings: FOV: 20 mm × 20 mm; slice thickness: 3 mm; matrix size: 256 × 256 pixels; number of averages: 20; TE: 8.1 ms; number of echos: 128; TR: 2000 ms; total scan time: 2 h 51 m; SW: 50 kHz. Emphasis was placed on maximizing the spatial resolution and the signal to noise ratio of the images, causing a degree of saturation as well as a degree of T_2_ weighting in the resulting amplitude map. The inversion recovery measurements were done using the following settings: FOV: 20 mm × 20 mm; slice thickness: 3 mm; matrix size 128 × 128; number of averages: 4; first echo: 4.6 ms; TE: 3.7 ms; inversion steps: 10 (50, 100, 200, 400, 800, 1000, 1500, 2000, 3000, 4000 ms); turbo factor: 8; TR: 4500 ms; SW: 50 KHz; scan time: 48 min. The datasets were fitted on a per pixel basis, using a mono-exponential decay function (van der Weerd et al., 2000), yielding maps of amplitude, T_2_ and T_1_ (Donker et al., 1997; Edzes et al., 1998). For the inversion recovery measurement, the resulting amplitude map was expressed in terms of VWC for which the 100% value was defined as the mean of the pixels in the center of the reference tubes.

After finishing the salinity experiment, three and a half months after the MRI study, plant 1 (Rm) was harvested and conserved in 50% ethanol. Transverse anatomical sections of the upper part of the hypocotyl were made with a sliding microtome (WSL Lab-Microtome, WSL, Birmensdorf, Switzerland). The obtained sections were placed on albumin-covered microscope slides, bleached and dried for 30 min at 70°C, then stained with Astra Blue (0.5%) and Safranin O (1%) for 3 min. Surplus stain was removed with 50 and 96% ethanol, progressively. The sections were embedded in xylol and Eukitt (BiOptica, Milan, Italy), covered with a cover glass and dried overnight. The cross section of the hypocotyl of plant 1 (Rm) was used as an anatomical reference for the MRI images.

### Stepwise 15-30-0‰ Salinity Treatments

The stepwise salinity experiment was done at the IBG-2: Plant Sciences Institute of the Forschungszentrum Jülich (Jülich, Germany). Prior to the experiment, the plants were grown in a climate chamber for an acclimatization period of 3 weeks (air temperature: 24–26°C, day–night variation 2°C; relative air humidity: ca. 75%; light: 145 μmol m^-2^ s^-1^ PAR; 12 h photoperiod) and watered with a 15‰ salinity solution. During the experiment, one plant per species was placed in different climate chambers to avoid possible crosstalk between the NMR sensors [air temperature: 26°C day and 24°C night; relative air humidity: ca. 65% (climate chamber 1) and ca. 75% (climate chamber 2); light: 185 μmol m^-2^ s^-1^ PAR (climate chamber 1) and 145 μmol m^-2^ s^-1^ PAR (climate chamber 2), 12 h photoperiod].

At the start of the experiment, the saplings were approximately 15 months-old. Plant growth was monitored weekly by recording the height of the plant and the hypocotyl diameter at 10 cm above the soil, by counting the number of leaves and by calculating the total leaf area after measuring individual leaf area of all leaves by tracing the outline on paper. The stomatal conductance was measured weekly using a SC1-Porometer (Decagon Devices, Pullman, WA, USA; four leaves per plant, three measurements per leaf).

Each plant (*N* = 4) was subjected to three salinity treatments: (i) 6–7 days at 15‰ (salinity at which the plants were grown), (ii) 5 days at 30‰, and (iii) 5 days at 0–5‰ salinity. To carry out these treatments, the pots with plants were placed in a water-filled bucket. Non-iodized salt was used to increase salinity. The salinity of the interstitial water in the soil was adjusted by repeatedly rinsing with large quantities of saline or freshwater until the salinity remained constant at the desired value. The water and soil water salinity was measured daily using a handheld refractometer (0–100‰, Erma, Tokyo, Japan), after pipetting the water or extracting it from the bottom of a plastic tube buried in the soil, and adjusted when needed.

### Mobile NMR Measurements

The water contents of the hypocotyls and leaves of the plants were continuously and non-invasively measured by means of novel mobile NMR sensors (Windt et al., 2011; Windt and Blumler, 2015; **Figure 1D**). Two sensors were used to monitor the hypocotyls of plant 1 (Rm) and 3 (Bg), and a leaf of plant 2 (Rm) and 4 (Bg). The NMR sensors consisted of C-shaped permanent magnets with a field strength of 0.235 T over an air gap of 37 mm. For the hypocotyl the magnet was fitted with a 13 turn, 20 mm (inner diameter, i.d.) solenoidal RF coil, hand-wound around the hypocotyl with the help of a Teflon mold. For leaves it was fitted with a 13 turn, 15 mm (i.d.) coil. In order to insert the leaf it was first rolled up and then inserted into the coil, effectively keeping the whole leaf in the dark for the duration of the experiment. For both plants, a representative leaf was selected for the measurements.

The NMR sensor was driven with a Magritek Kea II spectrometer (Magritek, Wellington, New Zealand) with a standard 100 W internal RF amplifier. To avoid temperature dependent modulations of signal amplification by the spectrometer, the spectrometer was placed in a temperature controlled isolated housing, capable of keeping spectrometer temperature constant to ±1°C.

T_2_ relaxometry was performed using a CPMG sequence and the following settings for both hypocotyl and leaf: TR 5 s; repetitions: 128; number of echoes: 4000; TE: 400 μs; SW: 200 kHz; pulse lengths 16 μs (leaf) and 8 μs (hypocotyl), Total scan time: 9 min per time point. Pulse amplitude varied to achieve excitation and refocusing.

To allow for real time, unsupervised data processing, no attempt was made to fit the multi-exponential T_2_ relaxation curve. Instead, all echoes between 0 and 25 ms were averaged. This maximizes signal to noise and avoids fitting errors that might otherwise occur when dealing with plant parts that may give rise to complex and changing multi-exponential T_2_ relaxation behavior, such as expected in the living hypocotyl and leaf samples measured under changing environmental conditions (Gultekin and Gore, 2005; Van As, 2007). See Appendix S1 for detailed information regarding the method and calibration.

The temperature of the saplings in the NMR sensor was continuously monitored. The slight changes in signal amplitude that would result from temperature – dependent changes in the Boltzmann equilibrium and minor changes in the resistance of the RF assembly were corrected by applying a correction factor of -0.44% per °C. The correction factor was determined independently by placing the NMR sensor together with a reference sample in a temperature controlled environment. The corrected NMR amplitude then scales linearly with the amount of liquid water inside the sensitive volume of the RF coil in the NMR probe head. Water content measured by means of the NMR sensor thus is expressed as WC_coil_, the amount of water detected in the sensitive volume of the NMR RF coil.

### Volume and Water Content of Hypocotyl and Leaves

During the stepwise salinity experiment, the total water content and changes in VWC were calculated from WC_coil_ and total organ volume. We thus assume that the water content in the middle of the hypocotyl or in the middle of the leaf closely matches the water content in the rest of the organ. Hypocotyl and total leaf volume were determined on the basis of measurements done at the start of the salinity experiment. Total hypocotyl volume was approximated using the truncated cone as:

(1) $$V\,=(1/3)\pi \left(r_{1}^{2}\,+r_{1}r_{2}+r_{2}^{2}\right)h\,\;$$

where *r*_1_ is the radius of the lowest part of the hypocotyl, *r*_2_ the radius of the highest part of the hypocotyl and *h* the length of the hypocotyl. Leaf surface measurements, obtained by tracing the leaves outlines on grid paper, were used to estimate the total leaf water content. The average leaf thickness was assumed to be the same for all leaves. At the end of the experiment the total fresh- and dry weights were measured gravimetrically. Fresh weight was measured directly after harvesting, dry weight after drying at 70°C for 48 h.

The NMR-based water content measurements were used to determine four further parameters:

(i) ΔWC_long term maximum_, here defined as the largest difference between the night values of two consecutive treatments, (ii) ΔWC_diurnal maximum_, the difference between the maximum values of two consecutive nights within the same treatment, (iii) ΔWC_step_, the maximum difference between the night value before and the night after the salinity step and (iv) ΔWC_day-night_, defined as the difference between the maximum night value and minimum day value of the same day (24 h).

### Dendrometer Measurements

In the absence of additional NMR sensors to measure water content directly, dendrometers were used as a proxy (De Swaef et al., 2015 and references therein). LVDTs (root and aquatic plant dendrometer DRO, Ecomatik, Dachau/Munich, Germany; sensor range 11 mm, resolution 2.6 μm) were placed on the hypocotyl at mid-height (**Figure 1C**).

Leaf thickness variation was measured with a miniature displacement transducer (DF-5.0, Solartron Metrology, Leicester, England; sensor range 5 mm, resolution rated “infinite,” limited only by the controlling hardware). The sensor was placed on the adaxial surface of one young- or middle-aged leaf; the dendrometer was held by a custom-built support (**Figure 1D**). To minimize the pressure on the leaf surface, the sensor rod was not pressed onto the leaf by means of a spring, but was simply placed on the leaf surface and held there by gravity alone.

The dendrometer data were logged and stored every 5 min (hypocotyl: HOBO U12 4-Channel External Data Logger – U12-006, Onset, MA, USA; leaf: CR1000, Campbell Scientific, Logan, UH, USA). The dendrometer data were analyzed using SAS v9.4 for Windows (Statistical Analysis System, SAS Institute, Cary, NC, USA). Hourly measurements were extracted as described by Deslauriers et al. (2011). A smoothing degree of 2 on a scale from 0 to 10 was used to avoid the loss of significant daily variation.

### Field Study

To compare our laboratory findings with observations in the field, we analyzed a dataset of dendrometer measurements on saplings of *R. mangle*, equally a Rhizophoraceae species with a growth habit and morphology closely matching those of *R. mucronata*. The study was conducted in the Avalon State Park (North Hutchinson Island, lagoon side, St. Lucie County, FL, USA; Feller et al., 2003, 2007). The dominant mangrove tree species in this forest is *Avicennia germinans* (L.) L. with scattered *Laguncularia racemosa* L. trees in the forest interior and individuals of *R. mangle* L. confined to the forest periphery (Feller et al., 2003). Three *R. mangle* saplings of similar age and size were selected, having six leaves and mid-height hypocotyl diameters of 7.70, 9.44, and 10.80 mm. The saplings were growing under the mangrove tree canopy in a sandy soil at a spot that was twice a day inundated by the tides so that the soil water salinity at this location was fluctuating around seawater salinity during periods without rainfall. The distance between the saplings was no more than 1.5 meters. On each sapling, a dendrometer (Root and aquatic Plant Dendrometer, DRO, Ecomatik, Dachau/Munich, Germany) was placed at hypocotyl mid-height. Radial hypocotyl changes were logged at a 10-min interval from 8 to 16 January 2014 with a resolution of 2.6 μm. Air temperature, RH and rainfall were registered during the study period (further details: Appendix S1).

## Results

For all plants the total water content of the hypocotyls was lower than the total water content of their leaves (total water content determined after harvest, **Table 1** – Organ water content). The hypocotyls of *R. mucronata* on average contained 45.3 g of water, versus 59.5 g for the leaves. For *B. gymnorrhiza* these numbers were 16.3 and 34.8 g, respectively (**Table 1** – Organ water content).

**Table 1:** Total organ fresh weight, dry weight and water content (WC), ΔWC long term maximum (difference between the night values of two consecutive treatments), ΔWC diurnal maximum (difference between the maximum values of two consecutive nights within the same treatment), ΔWC day–night (difference between the maximum night value and minimum day value of the same day) and ΔWC step (difference between the night value before and the night after the salinity step) in hypocotyl and leaf of *Rhizophora mucronata* and *Bruguiera gymnorrhiza* saplings during the step-wise salinity treatments.

		*Rhizophora mucronata*								*Bruguiera gymnorrhiza*							
		Plant 1 – Hypocotyl				Plant 2 – Leaves				Plant 3 – Hypocotyl				Plant 4 – Leaves			
Organ fresh weight	g	67.9				79.8g				23.3				42.6			
Organ dry weight	g	22.6				20.3				7.1				7.8			
Organ water content	g	45.3				59.5				16.2				34.8			

**Treatment**		**15‰**	**30‰**		**0–5‰**	**15‰**	**30‰**		**0–5‰**	**15‰**	**30‰**		**0–5‰**	**15‰**	**30‰**		**0–5‰**
ΔWC long term maximum	g	–	-1.49		3.67	–	-2.91		2.43	–	-0.58		0.91	–	-0.70		1.36
	%	–	3.3		8.1	–	4.9		4.1	–	3.6		5.6	–	2.0		3.9
ΔWC diurnal maximum	g	–	-0.50		0.40	–	-0.80		0.30	–	-0.15		0.10	–	–		–
	%	–	1.1		0.9	–	1.3		1.3	–	0.9		0.6	–	–		–
ΔWC day–night	g	0.66	1.41		0.20	1.96	4.56		3.27	0.17	0.29		0.19	0.71	1.84		1.98
	%	1.5	3.1		0.5	3.3	7.7		5.5	1.05	1.8		1.2	2.0	5.3		5.7

**Step treatment**		**15–30‰**		**30 to 0–5‰**		**15–30‰**		**30 to 0–5‰**		**15–30‰**		**30 to 0–5‰**		**15–30‰**		**30 to 0–5‰**	
ΔWC step	g	-0.40		1.20		-0.61		1.73		-0.11		0.55		–		–	
	%	0.9		2.6		1		1.7		0.7		3.4		–		–	

*Total organ fresh weight, dry weight and water contents of the hypocotyl and leaves were calculated on the basis of leaf and hypocotyl samples which were harvested after the experiment and analyzed gravimetrically. The dynamic water content variations were calculated on the basis of the WC_*coil*_ values as measured by NMR; – No data could be obtained.*

**FIGURE 2:**
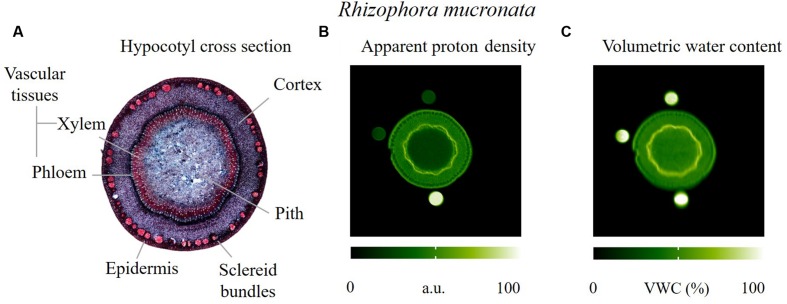
**Actual and virtual cross sections of the hypocotyl of a *R. mucronata* sapling, obtained by **(A)** optical microscopy, red coloration indicates lignified tissues, blue-purple coloration indicates tissues containing cellulose; **(B,C)** MRI parameter maps.** In image B the emphasis is put on maximizing the spatial resolution to more clearly resolve the sclereid bundles and to provide optimal anatomical contrast, albeit at the expense of moderate T_1_ weighting resulting from a combination of long ETs and short RTs. T_1_ weighting caused a loss of intensity in the pith and the top two reference tubes. Image C represents VWC.

We observed that the vascular tissues had the highest VWC with an average value of 68%, whereas, the epidermis, the cortex and the pith tissues had a VWC of 45–46% (**Figure 2C**). The sclereid bundles could not be fully resolved in the apparent proton density image (**Figure 2B**) nor in the VWC image (**Figure 2C**), but certainly appeared to possess a VWC that was lower than that found for the pith parenchyma. The low water contents in the pith and the sclereid bundles indicate the presence of relatively large amounts of air (**Figure 2A**).

### Stepwise Salinity Treatment

The stomatal conductance varied markedly in all plants in response to the salinity changes. It decreased at 30‰ salinity relative to the 15‰ treatment, and it increased at 0–5‰ salinity (**Table 2**). All plants grew over the duration of the experiment, with increases in height, diameter, and leaf area (**Table 2**). Only plant 3 (Bg) lost a leaf naturally, decreasing the total leaf area.

**Table 2:** Plant characteristics.

	*Rhizophora mucronata*				*Bruguiera gymnorrhiza*			
	Plant 1		Plant 2		Plant 3		Plant 4	
	Pre	Post	Pre	Post	Pre	Post	Pre	Post
Hypocotyl diameter (mm)	15.89	16.38	16.29	17.20	15.84	15.88	14.00	14.91
Height (cm)	60.50	59.50	71.00	75.00	60.00	61.00	48.00	50.50
Number of leaves	16	16	12	12	25	24	36	39
Total foliar area (cm^2^)	468.00	511.00	435.00	469.00	481.25	475.00	260.75	280.50
Stomatal conductance (mmol m^-2^ s^-1^ ± SD)								
Treatment 1 (15‰)	78.00 ± 31.77		140.73 ± 95.64		73.69 ± 37.29		153.42 ± 39.01	
Treatment 2 (30‰)	70.90 ± 26.56		76.06 ± 34.30		44.96 ± 20.91		80.10 ± 30.12	
Treatment 3 (0–5‰)	179.20 ± 23.56		129.51 ± 48.86		258.7 ± 34.53		250.80 ± 44.71	

*Hypocotyl diameter, plant height, number of leaves and total foliar area before (Pre) and after (Post) the stepwise salinity treatments and stomatal conductance during the three treatments in hypocotyl and leaves. The stomatal conductance values are calculated as the average of all measurements (three measurements per leaf, four leaves per plant).*

In the stepwise salinity treatment, we distinguish diurnal and long term responses, the first acting over the diurnal time course (hours), the second over the full duration of a salinity stage (days). In the next section the short and long term responses will be described in details.

#### Diurnal Response of Hypocotyls and Leaves

**FIGURE 3:**
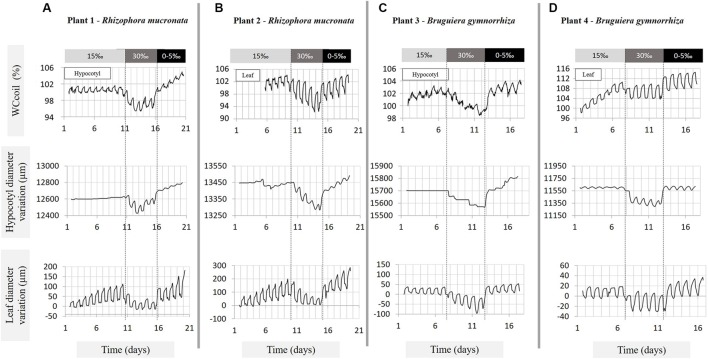
**Variations in hypocotyl and leaf water content (expressed as WC_coil_), in hypocotyl diameter and in leaf thickness, measured over 17–20 days under three salinity treatments (15, 30, 0–5‰ NaCl) on **(A)** plant 1 – *Rhizophora mucronata*, **(B)** plant 2 – *Rhizophora mucronata*, **(C)** plant 3 *– Bruguiera gymnorrhiza*, and **(D)** plant 4 *– Bruguiera gymnorrhiza*.** The salinity treatments are indicated by the bars above the graphs and delimited by the vertical dashed lines. The water content traces were measured by means of NMR sensors; the diameter and leaf thickness by means of dendrometers. The amplitude at the start of the NMR measurement is defined as 100%.

The diurnal fluctuations in leaf and hypocotyl VWC were strongly influenced by the salinity treatments (**Table 1**; **Figure 3**). After shifting to 30‰ salinity, the amplitude of the fluctuations increased by 65–71% in plant 1, 2 (Rm) and 4 (Bg), and by 42% in plant 3 (Bg), relative to the amplitudes at 15‰ salinity. At 30‰ salinity, in plants 1 and 2 (Rm) and plant 3 (Bg) the amount of water lost during the day was always larger than the amount replenished at night, causing a net water loss.

After transferral to 0–5‰ salinity, the diurnal fluctuations in the hypocotyl decreased, reaching values lower than or similar to the ones observed during the first treatment stage (15‰), whereas, in the leaf the amplitudes of the diurnal fluctuations were similar to the ones observed at 30‰ salinity or slightly lower, but larger than at 15‰ salinity (**Table 1** – ΔWC day–night). In plants 1 and 2 (Rm) and plant 3 (Bg) the water gain during the night was always higher than the water lost producing a daily net water gain (**Figure 3**).

The average amount of water lost and replenished during the day in the leaves was always larger than in the hypocotyl, regardless of the salinity treatment, for all plants and both species (**Table 1** – ΔWC day–night, **Figure 3**).

**FIGURE 4:**
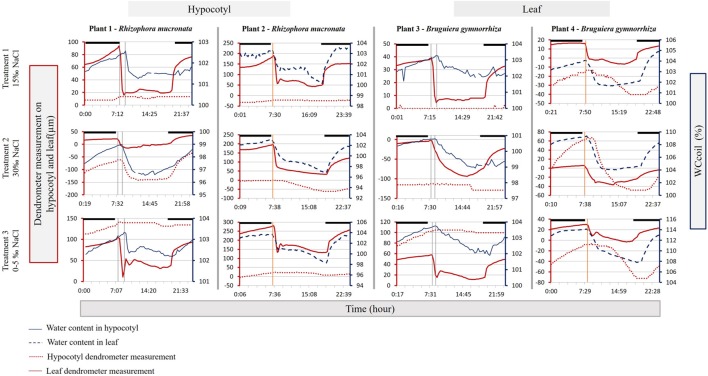
**Hypocotyl (solid blue line – right blue Y-axis) and leaf (dashed blue line – right blue Y-axis) water content (expressed as WC_coil_), hypocotyl diameter variation (dotted red line – left red Y-axis) and leaf thickness variation (solid red line – left red Y-axis) of one representative day for each plant and treatment (see **Figure 1**).** Darkness is indicated by black bars above the graphs. The vertical gray solid lines indicate the delay between the leaf- and the hypocotyl morning (light on) response. The vertical orange solid lines indicate the delay between the first response in leaf thickness and leaf WC_coil_.

During all stages of the experiment, the leaves reacted very quickly when the lights were turned on. At 7:00 the lights were turned on, resulting in marked decreases in VWC and leaf shrinkage within 15 to 45 min (**Figure 4**). In the hypocotyl a decrease in VWC was not observed until between 8:30 and 9:00, an average delay of about 80 ± 17 min (Rm) and 72 ± 17 min (Bg) after the leaves showed a first reaction (**Figure 4**). There was some variability in the delay, but it remained constant irrespective of the salinity treatment.

#### Long Term Response of Hypocotyls

Upon changing the salinity of the root medium from 15 to 30‰, the relative amount of water lost from the hypocotyl was similar in both species, amounting to 3.3% in plant 1 (Rm) and 3.6% in plant 3 (Bg). The depletion of water progressed gradually with a maximum daily net water loss of 1.1% in plant 1 (Rm) and 0.9% in plant 3 (Bg). After lowering the soil water salinity from 30 to 0–5‰ the increase in hypocotyl VWC was much larger than the decrease in VWC upon transfer from 15 to 30‰. In plant 1 (Rm), an increase of 8.1% was found, with a daily net maximum water gain of 0.9% whereas, in plant 3 (Bg) these values were 5.6 and 0.6% (**Table 1**; **Figures 3A,C**). While during the transition from the 15‰ to the 30‰ treatment the decrease of the VWC was slightly lower than the daily net maximum water loss (0.9%, plant 1, Rm) and 0.7% (plant 3, Bg), the increase in the VWC at the transition from the 30‰ to the 0–5‰ treatment was larger than the daily net maximum water gain (2.6%, plant 1, Rm) and 3.4% (plant 3, Bg; **Table 1**; **Figures 3A,C**).

The hypocotyl diameter variations almost perfectly mirrored the changes in VWC as measured by NMR, with the exception that the NMR measurement seemed to detect small changes more easily than did the dendrometers especially in the daily variations (**Figure 3**). At 30‰ salinity, a gradual decrease of between 100 and 130 μm in plants 1 and 2 (Rm; diameters of 12.60 and 13.45 mm, respectively), and in plant 3 (Bg; diameter 15.70 mm) was observed during the 5 days of treatment. Interestingly, plant 4 (Bg) did not show a gradual response, but instead exhibited a rapid decrease of ca. 200 μm (diameter: 11.60 mm) during the first day of treatment upon transferral from 15 to 30‰, and stayed at that level for the entire duration of the treatment. On transferral from 30 to 0–5‰, the amplitude of the hypocotyl swelling was similar for plants 1 and 2 (Rm) and plant 3 (Bg), gradually reaching values between 175 and 230 μm after 5 days of treatment in 0–5‰, whereas, plant 4 showed a rapid increase, reaching its new diameter value overnight (**Figure 3**).

#### Long Term Response of Leaves

Upon transferral from 15 to 30‰ salinity, the percentage decrease of leaf VWC was more than double in plant 2 (Rm) than in plant 4 (Bg; **Table 1** – ΔWC long term maximum, **Figures 3B,D**) while the transferral to 0–5‰ salinity induced a similar increase of the % VWC in the leaves of both plants. In plant 2 (Rm) the decrease and increase of VWC induced by the change in salinity was gradual, while in the leaf of plant 4 (Bg) the change in VWC occurred rapidly during the first day of treatment (**Table 1**; **Figures 3B,D**). The increase in leaf VWC observed in the transition day (from 30 to 0–5‰) and in the 0–5‰ treatment, was larger than the decrease observed in the transition day from 15 to 30‰ and in the 30‰ salinity treatment of both plants (**Table 1** – ΔWC step, **Figures 3A,C**).

The leaf thickness variations closely mirrored the response in VWC as measured by NMR (**Figures 3B,D**). The change in the amplitude tended to be much larger in Rm (plants 1 and 2; 100–230 μm) than in Bg (plants 3 and 4; 17–35 μm), after the transferral from 15 to 30‰ as well as for the transferral from 30 to 0–5‰ salinity (**Figure 3**). Plant 4 (Bg) is the only sapling that showed a fast change in the first day of treatments, all other saplings exhibited gradual changes.

In order to be able to place all sensors on plant 4 (Bg), the NMR measurements had to be done on a young growing leaf, while a fully grown leaf just below was used for the dendrometer measurements. During the first treatment (15‰ NaCl), the growth of the leaf translated into a constant increase in the VWC (NMR data) that was absent in the dendrometer data (**Figure 3D**), in which the net leaf thickness did not change. This was not observed in plant 2 (Rm), as here it was possible to place both devices on mature leaves of similar age.

### Field Study

**FIGURE 5:**
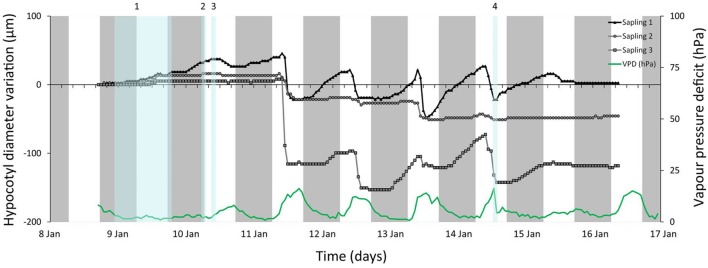
**Hourly hypocotyl diameter variations (relative to zero – left Y axis) and (VPD – right Y axis) for three *Rhizophora mangle* saplings in the Avalon State Park, FL, USA.** Gray bars indicate nights, blue bars indicate rainfall events. The starting diameter of each sapling is given between brackets in the legend. Amount of rain that has fallen during the corresponding rainfall events is: 1: 16.49 ml/cm^2^; 2: 0.03 ml/cm^2^; 3: 0.26 ml/cm^2^; 4: 0.20 ml/cm^2^.

*Rhizophora mangle* saplings 1 and 3, monitored in the Avalon State Park mangrove forest (FL, USA) exhibited comparable daily patterns of hypocotyl diameter variations during rainless days, i.e., late morning shrinkage and swelling in the early to late afternoon (**Figure 5**). The hypocotyl diameter variations of sapling 2 were less pronounced (**Figure 5**). During a day with continuous rainfall (9 January, 16.49 ml cm^-2^, **Figure 5**), all saplings showed an increase in hypocotyl diameter that continued for the next 2 days. On this day RH was high and air temperature low, causing an especially low VPD. After shorter rainfall events (10 and 14 January, **Figure 5**) a swelling or a reduction of hypocotyl shrinkage was observed as compared to drier days.

## Discussion

To estimate the amount of water that the hypocotyls and leaves of *R. mucronata* and *B. gymnorrhiza* saplings have available to buffer water potential changes in their abiotic environment, we submitted the saplings with three stepwise soil salinity treatments. These salinities can be regarded as typical of infrequently flooded area of the forest, where fast and drastic variations in soil water salinity are caused by spring and neap tides, freshwater input and rain events (Ball, 1988a; Tomlinson, 1994; Robert et al., 2009).

There was a reduction of stomatal conductance in the saplings when shifting from 15 to 30‰ NaCl and a marked increase in both parameters when shifting from 30 to 0–5‰ NaCl, reaching values higher than previously measured at 15‰. These observations are in agreement with prior studies that demonstrated a decrease in growth, stomatal conductance and photosynthesis in response to higher soil water salinities, and an increase in these physiological parameters at lower salinities (Parida et al., 2004; Hoppe-Speer et al., 2011).

### Water Storage Capacity of Hypocotyl and Leaves: Anatomy

To investigate what tissues in the hypocotyl could store water, MRI was done on the hypocotyl of *R. mucronata* specimens. The results showed evidence of both the original functions of the hypocotyl as a buoy (relatively high air content), and also demonstrated the presence of significant amounts of water. In the *R. mucronata* hypocotyl the vascular tissues showed a higher water content than the parenchymatous tissues (cortex and pith), but as the latter tissues make up the largest part of the hypocotyl by far, their water storage capacity should not be underestimated. In trees, the parenchyma is commonly associated with water storage (Morris et al., 2015; Pfautsch et al., 2015a). The hypocotyl reflects its function as a float during the dispersal phase of the propagule, but contains sufficient water storage tissues to be involved in water buffering once the young mangrove saplings are settled. The high air content of the hypocotyl could be valuable as an oxygen reserve during periods of inundation.

At approximately 15 months of age, the leaves of all mangrove saplings of both species under study had a higher cumulative water content than the hypocotyl, their VWC was 4–5% in response to changes in soil water salinity. The leaves of both species have a thick hypodermis and thick mesophyll layers, giving them a succulent appearance (Tomlinson, 1994; Das, 1999) and a large water storage capacity (Das, 1999; Suárez and Sobrado, 2000). *R. mucronata* leaves have a multilayer hypodermis, whereas, in *B. gymnorrhiza* it consists of a single layer of cells. This might explain why *R. mucronata* exhibited larger variations in leaf water content and thickness than *B. gymnorrhiza*. The differences in leaf thickness variations between the two species might also be influenced by the presence of branched fiber-sclereids in the mesophyll of *R. mucronata*, a feature absent in *B. gymnorrhiza*. Branched fiber-sclereids give mechanical support under low turgor conditions (Tomlinson, 1994; Das, 1999) and may thus physically limit the shrinkage of the leaf and help it preserve its structural stability when turgor is low.

### Long and Short Term Buffering

Shrinkage and swelling were observed in response to stepwise salinity changes. The diameter variations closely mirrored the changes in VWC, validating the use of the dendrometers as a proxy to observe changes in water content in leaf and hypocotyl. The NMR sensor detects small changes compare to the dendrometers.

Short term water content fluctuations, ΔWC_day-night_, were higher in the leaves than in the hypocotyl at all salinities, both in absolute and in relative terms. At 30‰ salinity, ΔWC_day-night_ increased by at least 50% relative to the volume withdrawn at 15‰ salinity. Upon shifting from 30 to 0–5‰, ΔWC_day-night_ decreased again in leaves and hypocotyls, but it tended to remain higher in leaves compared to native salinity (15‰). The fact that this daily pattern could also be observed at 15‰ salinity, the condition at which the plants were grown, as well as at 0–5‰ salinity, suggests that water storage capacity does not only have a role under adverse conditions but that it also helps to deal with mild daily variations.

The short term diurnal water content fluctuations were superimposed on much slower, long term responses of water loss at 30‰ and refilling at 0–5‰. In total, the maximum amount of water lost for the hypocotyl was 6.2% for *R. mucronata* and 4.8% for *B. gymnorrhiza*, against 11.7% for the leaves of *R. mucronata* and 7.3% for *B. gymnorrhiza*. Only plant 4 (Bg) did not show a gradual release of water during the salinity treatments, possibly due to its small size or to a damaged root. The latter cause might be the most likely one. *B. gymnorrhiza* has thick, brittle roots that sometimes grow out of the bottom of the pot. It is conceivable that during the experimental treatments one of these roots got damaged, thus temporarily damaging the root barrier and opening the xylem to the root environment. This way the plant would not be able to slowly use up its cumulative water storage pools, but in absence of a semipermeable barrier much more quickly reach equilibrium with the water status of the rhizosphere through direct exchange.

The long term responses indicate that water storage in hypocotyl and leaves does not contribute to short term buffering only. Similar patterns have been found in baobab trees (*Adansonia* spp. L.), in which stem stored-water usage has a major role during longer-term water deficits (Chapotin et al., 2006). Surprisingly, at 15 months of age, in all specimens of both species and under all treatments the water storage capacity of the leaves was found to be larger than that of the hypocotyl. This indicates not only that the leaves of both species add considerably to their water storage capacity, but also that at younger ages, when only few leaves have developed, the hypocotyl is likely to contain the bulk of the stored water in the sapling. At early developmental stages, the availability of water storage capacity might be even more important than later stages, as suggested by Smith and Snedaker (1995). We thus conclude that the role of the young hypocotyl after settlement is not only restricted to the storage of nutrients and air, which are the same functions as the structure had during hydrochory, but also includes the storage of water after the seedling has settled: a function that at first glance would seem to contradict its previous function as a float.

Under the relatively mild challenges that were imposed in the current study, the absolute amount of water released by the short and long term buffering responses was not very large. It may, however, still be sufficient to give the sapling a competitive edge. Evidence to this effect comes from sapling and mature trees. Studies done on five species of tropical adult trees showed that species with greater storage capacity maintained their maximum rates of transpiration for a substantially longer fraction of the day than trees with a smaller storage capacity, demonstrating how relatively small volumes of water withdrawn from the internal tissues can positively influence the carbon balance of a tree (Goldstein et al., 1998; Zweifel and Häsler, 2001).

While the depletion of the water storage pools of the saplings took several days, the replenishment of water storage pools in leaves and hypocotyls occurred very fast when transferred from 30 to 0–5‰. This confirms that, as has been observed for radial growth in adult *Avicennia marina* trees (Robert et al., 2014; Santini et al., 2015), young mangrove trees take advantage of short spells of favorable conditions to quickly replenish their water reserves. The ability to recover after salt stress and to make the most of brief periods of low salinity may not only be key to species survival (Hale and Orcutt, 2000; Bompy et al., 2014a), may also strongly influence mangrove forest structure as it affects which seedlings survive at what positions along gradients of height, salinity, likelihood of freshwater input, and tidal exposure (Ball, 2002).

### Time Delayed Responses

A surprising result was that even in small saplings, buffering responses are associated with considerable time lags. In both species and during all salinity treatments, light exposed leaves almost instantly lost water or shrunk as soon as the lights were turned on. The hypocotyl, on the other hand, did not exhibit loss of water or shrinkage until 72 (Bg) to 80 min (Rm) later. A much shorter time lag of 5–15 min was measured between a leaf exposed to the light, and another leaf on the same plant in the dark inside the RF coil of the NMR sensor. Due to its proximity, the leaf inside the RF coil probably provided stored water to the transpiring leaves, an hour before the hypocotyl did.

The occurrence of time lags in diameter changes among different stem heights, or between stem, crown or leaves, has been well-documented in adult trees in response to abiotic stimuli (Goldstein et al., 1998; Zweifel and Häsler, 2001; Zweifel et al., 2001; Sevanto et al., 2002) even when the plants were well-watered (Zweifel et al., 2000). In Scots pine (*Pinus sylvestris* L.) Sevanto et al. (2002) measured a time lag between the “total” stem (meant as xylem and bark) and the xylem (without bark) that varied from 30 to 110 min, proving that the bark tissues serve as a water reserve to replace the water lost during transpiration.

As far as we are aware, such delayed responses, typically associated with storage capacity and buffering, have not been reported for saplings. This signifies that the leaves and hypocotyls do buffer sudden changes in the abiotic environment of the sapling.

### Field Conditions

The observed rapid swelling of the sapling hypocotyl tissues during the day with extensive rainfall, confirmed that mangrove saplings have the ability to rapidly take benefit of freshwater input. In the absence of the experimental abrupt light on or light off events of the laboratory, the diurnal pattern of swelling and shrinkage was less pronounced than in the laboratory, but closely mirrored the changes in VPD in the forest after the rain had stopped. The observation that also in *R. mangle* and under natural conditions the hypocotyl responds to changes in water potential and VPD, strengthens the view that stored water in the hypocotyl is mobilized, depleted and replenished as needed even under non-stress conditions in the field.

More field experiments are needed to clarify the exact role and dynamics of the water reserves in the different plant organs under natural conditions, considering periods of extreme change in salinity. It could also be interesting to verify whether foliar rain water uptake can play a role in directly feeding into the storage water capacity of the leaves, analogous to the foliar uptake of water in trees where sheer tree height poses an impedance to the transport of water taken up by the roots (Limm et al., 2009; Simonin et al., 2009; Ishii et al., 2014).

## Author Contributions

SL and ER: conception and design of the study, acquisition of data, analysis and interpretation of the data, drafting and revising of the manuscript. NT: acquisition of data, analysis and interpretation of the data, revising of the manuscript. AP, EG: acquisition of data, analysis of the data, revising of the manuscript. HVA: analysis and interpretation of the data and revising of the manuscript. NK: analysis and interpretation of the data and revising of the manuscript. CW: conception and design of the study, construction of the NMR sensors, acquisition of data, analysis and interpretation of the data, drafting and revising of the manuscript.

## Conflict of Interest Statement

The authors declare that the research was conducted in the absence of any commercial or financial relationships that could be construed as a potential conflict of interest. The reviewer AD and handling Editor declared their shared affiliation, and the handling Editor states that the process nevertheless met the standards of a fair and objective review.

## Abbreviations

Bg: *Bruguiera gymnorrhiza*
CPMG: Carr Purcell Meiboom Gill
ΔWC: delta water content
FOV: field of view
IDL: Interactive Data Language
LVDTs: linear variable displacement transducers
MRI: magnetic resonance imaging
NMR: nuclear magnetic resonance imaging
PAR: photosynthetically active radiation
RF: radio frequency
Rm: *Rhizophora mucronata*
SW: spectral width
TE: echo time
TR: repetition time
VPD: vapor pressure deficit
VWC: volumetric water content.
