# Supplementary material for: Rhizophoraceae Mangrove Saplings Use Hypocotyl and Leaf Water Storage Capacity to Cope with Soil Water Salinity Changes
Source: Front Plant Sci. 2016 Jun 27;7:895. doi: 10.3389/fpls.2016.00895 (PMC4921503; doi:10.3389/fpls.2016.00895)
Supplement: Supplementary file 1 [file Data_Sheet_1.DOCX]

Supplementary Material for

**Rhizophoraceae mangrove saplings use hypocotyl and leaf water storage capacity to cope with soil water salinity changes**

Silvia Lechthaler^1,2‡^, Elisabeth M. R. Robert^2,3‡^, Nathalie Tonné^2,3^, Alena Prusova^4^, Edo Gerkema^4^, Henk Van As^4^, Nico Koedam^2^ and Carel W. Windt^5,6^

^1^Department of Territorio e Sistemi Agro-Forestali, University of Padova, Viale dell'Università 16, Legnaro (Padova), I-35020, Italy

^2^Laboratory of Plant Biology and Nature Management, Vrije Universiteit Brussel, Pleinlaan 2, Brussel, B-1050, Belgium

^3^Laboratory of Wood Biology and Xylarium, Royal Museum for Central Africa (RMCA), Leuvensesteenweg 13, Tervuren, B-3080, Belgium

^4^Laboratory of Biophysics and Wageningen NMR Centre, Department of Agrotechnology & Food Sciences, Wageningen University, Dreijenlaan 3, P.O. Box 8128, Wageningen, NL-6700 ET, Netherlands

^5^IBG-2: Plant Sciences, Institute for Bio- and Geosciences, Forschungszentrum Jülich, Jülich, DE-52425, Germany

^6^Correspondence c.windt@fz-juelich.de

^‡^These authors contributed equally to the work.

**Supplementary Material: Appendix S1**

**NMR sensor: reference measurements**

To demonstrate that the amplitude of the NMR signal scales linearly with the volume of water inside the sensitive volume of the RF coil in the NMR probe head, three reference experiments were conducted. First, aliquots of 1 ml of a Ni(NO_3_)_2_ reference solution with a T_2_ of 200 ms were pipetted into a test tube with its bottom placed in the centre of the sensitive volume of the coil. Three measurements were done of every aliquot. Coil water content was subsequently plotted against NMR amplitude (Fig. S1A).

Second, to confirm the existence of a linear correlation between hypocotyl water content and NMR signal amplitude, hypocotyls of both species were harvested (plant 1, Rm and plant 3, Bg) and cut into 2 cm sections. The sections were weighed on an analytical scale (ENTRIS64-1S, Sartorius, USA), immediately placed in the NMR probe and measured, then put on the table top to dry until the last hypocotyl piece was measured; then weighed and again measured in the NMR sensor. The samples were cycled in the measurement routine until the hypocotyl sections had lost at least 10 % of their total weight. To obtain the dry weight of the samples the sections were dried overnight in an oven at 70 °C. Subsequently the water content (WC) of the samples was calculated.

Third, to confirm a linear correlationship between leaf WC and NMR signal amplitude, a similar procedure was followed. Eight leaf disks of 0.8 cm in diameter were collected for both plant 2 (Rm) and plant 4 (Bg), weighed on an analytical scale (ENTRIS64-1S, Sartorius, USA) and placed in the NMR probe immediately after weighing. After the NMR measurements the leaf disks were allowed to dry under a moderate flow of compressed air, while the next samples were measured. The sequence was repeated until the leaf sections had lost approximately 25 % of their weight. Afterwards the leaf samples were dried overnight in an oven at 70 °C to obtain the dry weight. Subsequently the WC of the samples was calculated.

The NMR amplitude was found to correlate well and scale linearly with probe (RF coil) water content (Fig. S1A), hypocotyl WC_coil_ (Fig. S1B) and leaf WC_coil_ (Fig. S1C). Due to differences between the coil diameters, receiver amplification settings and T_2_ relaxation characteristics of the different samples, the amplitude response per ml or mg of liquid water was not equal in absolute units, but can be assumed to be linear for the various samples for water content values in the range that was tested here.

**Field study -** Environmental data

The air temperature and relative air humidity were logged in a 10 minute interval using a HOBO U23 pro Temperature/Relative Humidity Data Logger (Onset, Bourne, MA, USA). Local rainfall data were obtained via a local commercial (Locher Environmental Technology, Port Charlotte, Florida, USA) and a self-made pluviometer. Additional rainfall data were obtained from the National Weather Service's weather station at the St. Lucie County International Airport in Fort Pierce (National Weather Service - NWS, National Oceanic and Atmospheric Administration (NOAA)), located 8 km south-west of the mangrove forest. NWS data were used to calculate the vapor pressure deficit (VPD) using the following formula:

$$VPD=\left( 1-(H*{100}^{-1} \right)*H)$$

where H is the saturated vapor pressure for the given temperature.

**Figure legends**

**Fig. S1.** NMR reference curves. (A) The NMR signal amplitude correlates linearly with the amount of water in the sensitive volume of the RF coil of the NMR sensor, here illustrated by pipetting 1 ml aliquots of a reference liquid in to a vessel that was placed in the NMR sensor. (B) The NMR amplitude scales linearly with hypocotyl water content, here confirmed by means of *Rhizophora mucronata* and *Bruguiera gymnorrhiza* hypocotyl samples that in between measurements were allowed to dry down. (C) The leaves of *Rhizophora mucronata* and *Bruguiera gymnorrhiza* have slightly different NMR signatures, necessitating the use of separate reference curves for the two. Per species, however, the leaf water content as measured in drying leaf disks again scaled linearly with NMR amplitude.
