# Supplementary figures and images for: Rhizophoraceae Mangrove Saplings Use Hypocotyl and Leaf Water Storage Capacity to Cope with Soil Water Salinity Changes
Source: Front Plant Sci. 2016 Jun 27;7:895. doi: 10.3389/fpls.2016.00895 (PMC4921503; doi:10.3389/fpls.2016.00895)

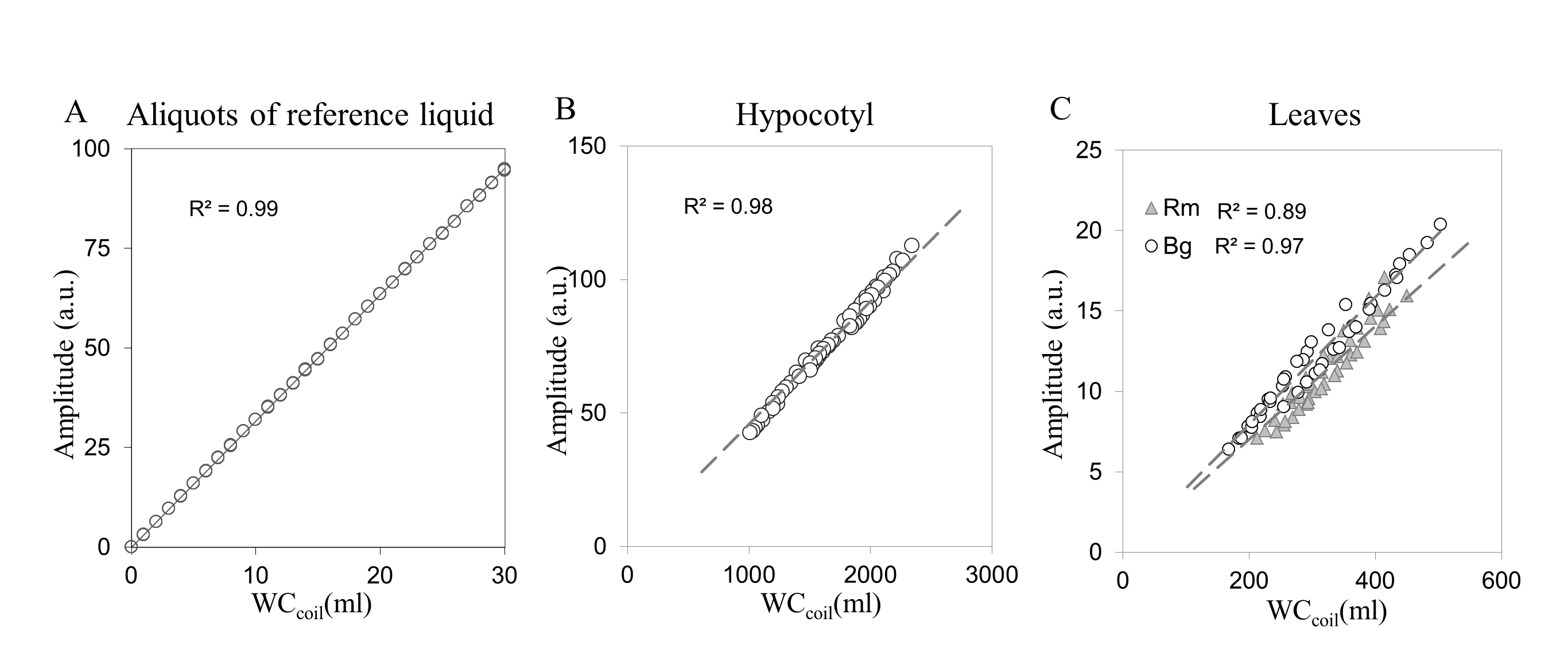

Supplement: Supplementary file 2 [file Image_1.JPEG]
